# The BioFire® RP2.1 Panel Did Not Identify Concurrent Respiratory Virus Infection in Adults with Variable SARS-CoV-2 Disease Severity and Infection Duration

**DOI:** 10.1155/2022/1378482

**Published:** 2022-08-08

**Authors:** Kendra M. Quicke, Bridget A. Baxter, Sophia Stromberg, Emily N. Gallichotte, Emily Fitzmeyer, Michael C. Young, Kristy L. Pabilonia, Nicole Ehrhart, Julie Dunn, Gregory D. Ebel, Elizabeth P. Ryan

**Affiliations:** ^1^Arthropod-Borne and Infectious Diseases Laboratory, Department of Microbiology, Immunology and Pathology, Colorado State University, Fort Collins 80526, CO, USA; ^2^Department of Environmental and Radiological Health Sciences, College of Veterinary Medicine and Biomedical Sciences, Colorado State University, Fort Collins 80523, CO, USA; ^3^Columbine Health Systems Center for Healthy Aging and Department of Clinical Sciences, Colorado State University, Fort Collins 80523, CO, USA; ^4^University of Colorado Health, Medical Center of the Rockies, Loveland 80538, CO, USA

## Abstract

SARS-CoV-2 emerged in 2019 and rapidly surged into a global pandemic. The rates of concurrent infection with other respiratory pathogens and the effects of possible coinfections on the severity of COVID-19 cases and the length of viral infection are not well defined. In this retrospective study, nasopharyngeal swab samples collected in Colorado between March 2020 and May 2021 from SARS-CoV-2 PCR-positive individuals were tested for a panel of 21 additional respiratory pathogens, including 17 viral and 4 bacterial pathogens. We detected significant positive correlations between levels of SARS-CoV-2 RNA and infectious virus titers for both cohorts, as well as a positive correlation between viral RNA levels and disease severity scores for one cohort. We hypothesized that severe COVID-19 cases and longer SARS-CoV-2 infections may be associated with concurrent respiratory infections. Only one individual exhibited evidence of a concurrent infection- SARS -CoV-2 and human rhinovirus/enterovirus- leading us to conclude that viral respiratory coinfections were uncommon during this time and thus not responsible for the variations in disease severity and infection duration observed in the two cohorts examined. Mask wearing and other public health measures were imposed in Colorado during the time of collection and likely contributed to low rates of coinfection.

## 1. Introduction

Infection with severe acute respiratory syndrome coronavirus 2 (SARS-CoV-2) causes coronavirus disease 2019 (COVID-19). Multiple reports document viral and bacterial infections occurring concurrently with SARS-CoV-2 [1–3]. However, the frequency of concurrent infections and the implications for COVID-19 are not well defined. For instance, there are conflicting reports on the frequency of SARS-CoV-2 coinfection with influenza viruses [3–6] which may be influenced by when samples were collected, with higher rates of coinfection occurring before mask mandates and other public health measures were implemented. One study found that coinfection of SARS-CoV-2 with influenza B resulted in more severe disease and overall poorer prognosis than coinfection with influenza A [5]. There are also a limited number of reports of coinfection with human rhinovirus (hRV) [2, 3, 7] and other endemic human coronaviruses (HCoV-229E, -HKU1, -NL63, and -OC43) [3, 7].

Studies of patients with respiratory coinfections documented more severe disease than those with a single infection [2, 8, 9]. Concurrent infections may enhance the inflammatory immune response in the lungs, resulting in worse disease [9, 10]. Alternatively, concurrent infections may effectively suppress immune responses, resulting in lengthier infections and reduced pathogen clearance [11, 12].

This retrospective study evaluated the extent of coinfection among SARS-CoV-2 infected individuals and whether concurrent infection of SARS-CoV-2 and other respiratory pathogens influences the overall severity and length of infection using the BioFire® platform with the Respiratory 2.1 (RP2.1) panel. Adults enrolled in two COVID-19 cohorts were included [13, 14]. Of the 193 nasopharyngeal samples tested, only one showed evidence of concurrent infection, suggesting that neither SARS-CoV-2 disease severity nor infection duration was significantly associated with concurrent infection with other respiratory viruses in these two US-based cohorts.

## 2. Materials and Methods

### 2.1. Cohort Recruitment and Selection

Cohort 1 individuals were from the Northern Colorado Coronavirus Biorepository (NoCo-COBIO) [13] and samples were collected between July 2020 and May 2021. Individuals had to have previously tested positive for SARS-CoV-2 infection via a PCR test of a nasopharyngeal swab to be considered for participation. This biorepository was approved by the Colorado State University Research Integrity and Compliance Review Office Institutional Review Board (IRB; protocol #2105/20–10063H), the University of Colorado Health IRB (Colorado Multiple 20–6043), and registered with ClinicalTrials.gov (NCT04603677). All enrolled participants provided written informed consent.

Cohort 2 individuals were from a longitudinal SARS-CoV-2 surveillance study of staff in Colorado long-term care facilities (LTCFs) [14] with samples collected between March and June 2020. Human subjects research was approved by the Colorado State University IRB (protocol #20–10057H). All participants provided written informed consent.

### 2.2. Nasopharyngeal (NP) Swab Collection

NP swabs were collected with latticed nasal swabs (Resolution Medical) by trained personnel, placed in viral transport medium (VTM; sterile Hanks balanced salt solution (HBSS), FBS (2%), gentamicin (50 mg/ml), and amphotericin B/fungizone (250 ug/ml)), and kept on ice during transportation. Aliquots of NP swab material in VTM were stored at −80°C. Samples were deidentified and labeled with unique study code identifiers. Samples were thawed for BioFire® testing.

### 2.3. RNA Extraction and qRT-PCR

Before freezing, RNA was extracted from 200 ul NP swab material in VTM using Omega Mag-Bind Viral DNA/RNA 96 kits on a KingFisher Flex magnetic particle processor as per manufacturer instructions. Reverse transcription and PCR were performed using EXPRESS One-Step SuperScript qRT-PCR kits (ThermoFisher Scientific) on a QuantStudio3 (AppliedBiosystems) as per manufacturer instructions. The SARS-CoV-2 N1 primer/probe set was obtained from IDT, and N1 RNA standards used to determine viral load were provided by Dr. Nathan Grubaugh of Yale University.

### 2.4. Plaque Assay

Plaque assays for infectious virus were performed using standard methods. Briefly, NP swab material in VTM was serially diluted and applied to African Green Monkey Kidney (Vero) cells (ATCC CCL-81) for one hour. Cells were overlaid with tragacanth medium. After a two-day incubation, overlay was removed, cells were stained with a crystal violet solution (ethanol (30%) and crystal violet (0.1%)), and plaques were counted manually.

### 2.5. Disease Severity Scoring

Cohort 1 contains hospitalized and nonhospitalized participants. COVID-19 disease severity was categorized using the Yale Impact Score based on oxygen requirements during the acute phase of illness: mild (no oxygen required), moderate (1–5 L/min oxygen required), and severe (>5 L/min oxygen required) [15].

For cohort 2, symptom severity was determined by a survey reliant on self-reporting of 11 symptoms. Disease severity scores were calculated as the average of these 11 individual symptom severity scores (0 = no symptom, 1 = mild, 2 = moderate, 3 = severe) [14]. To our knowledge, none of these participants were hospitalized due to SARS-CoV-2 infection.

### 2.6. BioFire® Testing

Samples were run on the BioFire® FilmArray® Torch system using the BioFire® Respiratory 2.1 (RP2.1) Panel (EUA) as per manufacturer instructions. NP samples in VTM are compatible with this system. The BioFire® RP2.1 panel detects 18 viruses and 4 bacterial species associated with respiratory illness, including SARS-CoV-2 [16]. Positive and negative controls are included with the panel and were run for each new kit. Positive control runs successfully detected all 22 targets in the RP2.1 panel every time.

### 2.7. Statistical Analysis

Correlations were determined using a nonparametric Spearman correlation (r_S_) test (2-tailed, *α* = 0.05). Comparisons between pneumonia and nonpneumonia patients were performed using a nonparametric Mann–Whitney test (2-tailed, *α* = 0.05). All statistical analyses were performed in GraphPad Prism 8 (v8.4.3).

## 3. Results

### 3.1. Cohort Characteristics

Two cohorts of SARS-CoV-2-positive individuals were assessed in this study. Cohort 1 included individuals with a SARS-CoV-2-positive nasopharyngeal (NP) swab PCR test [13]. Participants (*n* = 122) were aged 18–85 years (mean 51) and BMI ranged from 19.4 to 64.5 (mean 31.6). The majority were females (55.7%) with 80.3% of participants identifying as Caucasian, 17.2% as Hispanic/Latino, 1.6% as Asian, and 0.8% as Native American (Table 1).

Cohort 2 included select samples from longitudinal surveillance of staff at LTCFs in Colorado [14] (*n* = 28). They were aged 17–70 years (mean 42) and were predominantly female (82.1%). BMIs ranged from 21.0 to 43.9 (mean 28.0; Table 1). No race data were collected. Weekly NP swabs were tested for SARS-CoV-2 viral RNA (vRNA) by qPCR.

### 3.2. Viral Load Correlations with Viral Titer and Disease Severity

**Figure 1:**
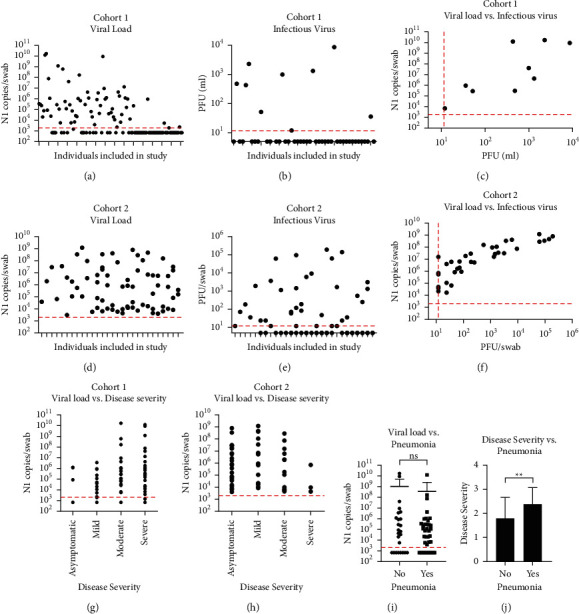
Viral load correlations with infectious viral titer and disease severity. (a, d) Viral loads for cohorts 1 and 2, determined by qRT-PCR as SARS-CoV-2 N1 RNA copies per NP swab. The red dashed line indicates limit of detection at 2,273.5 N1 copies/swab. (b, e) Infectious virus titers for cohorts 1 and 2, determined by plaque assay as plaque-forming units (PFU) per ml. The red dashed line indicates limit of detection at 12 PFU/ml. (c, f) Correlation between viral load and infectious virus titer for cohorts 1 and 2. Statistical correlation was determined by the Spearman nonparametric test. (g, h) Correlation between viral loads and COVID-19 severity for cohorts 1 and 2. Statistical correlation was determined by the Spearman nonparametric test. The red dashed line indicates the limit of detection. (i, j) Cohort 1 viral load and disease severity in patients with pneumonia compared to patients without pneumonia. Statistical association was determined by the Mann–Whitney nonparametric test (^*∗∗*^*p* < 0.01). The red dashed line indicates limit of detection.

In cohort 1, 122 PCR-positive samples from 122 individuals were assessed for infectious virus. Viral loads, calculated as SARS-CoV-2 N1 RNA copies, and infectious viral titers varied greatly among individuals (Figures 1(a)-1(b)). A positive PCR result did not guarantee detection of infectious virus; however, there is a positive relationship between viral load and viral titer by Spearman correlation (r_S_ = 0.4789, *p*=0.0007, Figure 1(c)).

In cohort 2, 71 PCR-positive samples from 28 individuals were tested for infectious virus. As with cohort 1, viral loads and viral titers varied (Figures 1(d)–1(e)). The original study found that viral load was positively correlated with infectious virus titers [14], and this held true for the smaller subset used herein as well (r_S_ = 0.8075, *p* < 0.0001, Figure 1(f)).

We observed a significant positive correlation between viral load and disease severity in cohort 1 (r_S_ = 0.3315, *p* = 0.0002, Figure 1(g)), but not cohort 2 (r_S_ = -0.1135, *p* = 0.3839, Figure 1(h)). The results from cohort 2 reflect findings from the larger LTCF study [14]. For cohort 1, the incidence of pneumonia was also recorded. Pneumonia cases (*n* = 37) were identified in the hospital setting via electronic medical record documentation of a positive chest X-ray. Notably, we found no significant difference in viral loads between individuals with pneumonia and those without (*p* = 0.7405; Figure 1(i)). However, the adults who had pneumonia experienced higher COVID-19 disease severity scores when compared to those without pneumonia (*p* = 0.0062; Figure 1(j)).

### 3.3. Disease Severity Does Not Correspond to Concurrent Infection with Other Respiratory Viruses Using the BioFire RP2.1 Panel

Participants in both cohorts exhibited a range of asymptomatic to severe diseases (Table 2). We hypothesized that more severe COVID-19 cases would be associated with the presence of additional respiratory pathogens, while asymptomatic and mild cases would not. We tested NP samples from 145 individuals from both cohorts.

No samples from cohort 1 were positive for the other respiratory pathogens in the BioFire® RP2.1 panel (Table 2). Additionally, only one sample from cohort 2 tested positive for an additional pathogen, human rhinovirus/enterovirus (Table 2, Figure S1). This sample was from a mild case of COVID-19 and no other SARS-CoV-2-positive NP swabs from this individual tested positive for human rhinovirus/enterovirus. Thus, we conclude that the variation in COVID-19 disease severity observed in these two cohorts is not the result of concurrent infection with one of the other common respiratory viruses included in the BioFire® RP2.1 panel.

There were no cohort 1 participants and there was only one cohort 2 individual who exhibited concurrent infection. Therefore, we concluded that SARS-CoV-2 infection did not predispose patients to coinfection with other common respiratory viruses during their hospital stay during the time period when stricting public health measures were in place, such as mask-wearing and stay at home/safer at home mandates.

### 3.4. Longer SARS-CoV-2 Infections Do Not Correspond to Concurrent Infection with Other Respiratory Viruses

During the LTCF surveillance study, SARS-CoV-2 vRNA was detectable for between one and four consecutive weeks [14]. Additionally, some individuals appeared to have recrudescence of vRNA after a period of negative test results. We hypothesized that longer SARS-CoV-2 infections and recrudescent infections would be associated with the presence of additional respiratory pathogens, while shorter infections would not. We tested all SARS-CoV-2 vRNA-positive samples from individuals with long (4+ weeks) and short (1-2 weeks) infection courses and those that exhibited recrudescent infections. We limited short infection samples to those from individuals with detectable infectious viruses.

Only one sample contained evidence of concurrent infection with another common respiratory pathogen (Table 3). The sample was the first NP swab taken from an individual with a long infection course and contained viral RNA from a human rhinovirus/enterovirus (Figure S1). No evidence of concurrent infection was detected in any subsequent SARS-CoV-2-positive samples from this individual. Thus, in cohort 2, longer SARS-CoV-2 infection courses were not associated with concurrent infection with any of the common respiratory viruses included in the BioFire® RP2.1 panel.

We also observed differences in infection duration within cohort 1. The time between the individuals' initial PCR test and the first NoCo-COBIO NP swab varied from less than one week to greater than five weeks. We defined a short infection as a positive initial PCR test followed by a negative PCR test at the first NoCo-COBIO visit when sample collection occurred within two weeks. A long infection was defined as a positive initial PCR test followed by another positive PCR test at the first NoCo-COBIO visit when sample collection occurred four or more weeks after initial PCR testing. No NP swabs contained evidence of additional pathogens, supporting the conclusion that length of infection is not associated with concurrent infection with any of the viruses included in the BioFire® RP2.1 panel (Table 3).

## 4. Discussion

Simultaneous infection with multiple respiratory pathogens can result in more severe disease due to the enhancement of inflammatory immune responses in target tissues [2, 9]. Concurrent infections can also compound the suppression of other immune responses, increasing the time it takes to clear the pathogens [9, 10]. This retrospective analysis of 193 samples from 150 individuals via the BioFire® Respiratory (RP2.1) panel [16] revealed only one individual who tested positive for SARS-CoV-2 and another respiratory pathogen. Thus, in these two cohorts, variations in SARS-CoV-2 disease severity and infection duration were not associated with concurrent infection with other common respiratory viruses included in the BioFire® RP2.1 panel. Notably, while cohort 1 viral loads were positively correlated with disease severity, this correlation was not the result of coinfection. We did not observe a correlation between viral load and disease severity in participants from cohort 2. This may be because individuals in cohort 2 experienced milder disease overall with only one case scored as severe (Table 2). Future studies should also consider testing samples from hospitalized patients at multiple time points during their hospital stay to determine their risk for developing concurrent infections with SARS-CoV-2.

The BioFire® RP2.1 panel detects 18 viral and 4 bacterial pathogens, including SARS-CoV-2, that commonly cause respiratory illness [16]. 59% of individuals in this study reported pneumonia, and it is possible they were infected with another pathogen not included in this panel. Indeed, one study found the most common concurrent infections with SARS-CoV-2 were *Streptococcus pneumoniae*, *Klebsiella pneumoniae*, and *Haemophilus influenzae* [2], which are not included in the BioFire® RP2.1 panel. BioFire® produces a pneumonia diagnostic panel that detects many of the same viruses as the Respiratory (RP2.1) panel, though does not include SARS-CoV-2, but contains 16 additional bacterial targets [17]. This panel is compatible with either bronchoalveolar lavage (BAL) or sputum samples, neither of which were collected for the two cohorts used in our retrospective study. Further investigations into the implications of concurrent infection in COVID-19 patients should consider collecting diverse sample types to expand the range of detectable pathogens.

We suspect rates of respiratory virus coinfection were negatively impacted by public health measures and mask mandates imposed in Colorado during the time of sample collection [18]. From March to April 2020, Colorado was under a stay at home order, which included strict public health mandates requiring mask-wearing and social distancing, limiting gatherings to members of the same household, and limiting business customer and employee capacities. From May to August 2020, under a safer at home order, some of the stricter measures were lifted, but mask-wearing and social distancing requirements and limits on public gatherings remained. With the arrival of SARS-CoV-2 vaccines, the mask mandate was lifted for vaccinated individuals in May 2021. Mask compliance for these two cohorts was not recorded, but this study suggests mask-wearing keeps coinfection rates low to nonexistent in people infected with SARS-CoV-2.

To cite a specific example, there was a notable reduction in the number of influenza cases across the United States in 2020 [19, 20]. Masks seem to effectively hinder the spread of influenza which would subsequently reduce the number of concurrent infections observed with SARS-CoV-2. Indeed, the variability in the rate of this particular coinfection appears to be somewhat dependent on when samples were collected, with higher rates observed before mask mandates, and other public health measures were implemented [3–6].

## 5. Conclusions

In conclusion, concurrent infection of SARS-CoV-2 with the other common respiratory viruses included in the BioFire® RP2.1 panel was not an important factor in determining variations in disease severity or infection duration in two Colorado-based cohorts sampled between March 2020 and May 2021. Mask-wearing, social distancing, and other public health measures may have effectively reduced the spread of other respiratory pathogens even if they did not prevent transmission of SARS-CoV-2, a testament to the heightened virulence of this virus.

## Figures and Tables

**Table 1:** Cohort demographics.

Characteristics		Cohort 1 (*n* = 122)	Cohort 2^1,2^ (*n* = 28)	*P* value^4^
Age		51 (18–85)	42 (17–70)	0.0094
BMI		31.6 (19.4–64.5)	28.0 (21.0–43.9)	0.0807
Sex	Male Female	44.3% (54/122)	14.3% (4/28)	—
		55.7% (68/122)	82.1% (23/28)	—
Race	Caucasian Hispanic/Latino Asian Native American	80.3% (98/122)	*N/A* ^*3*^	—
		17.2% (21/122)	*N/A* ^*3*^	—
		1.6% (2/122)	*N/A* ^*3*^	—
		0.8% (1/122)	*N/A* ^*3*^	—

^1^Seven individuals did not report BMI (body mass index). ^2^One individual did not report sex. ^3^Race data were not collected for this cohort. ^4^Unpaired *t*-test, 2-tailed, *α* = 0.05.

**Table 2:** COVID-19 severity and concurrent respiratory infections.

			BioFire® testing			
Disease severity	# of individuals		SARS-CoV-2 (+)		Other respiratory pathogens (+)	
	Cohort 1 (*n* = 122)	Cohort 2^1^ (*n* = 23)	Cohort 1	Cohort 2	Cohort 1	Cohort 2
Asymptomatic	6 (4.9%)	9 (39.1%)	3 (50.0%)	8 (88.9%)	0 (0%)	0 (0%)
Mild	48 (39.3%)	7 (30.4%)	15 (31.3%)	7 (100%)	0 (0%)	1 (14.3%)
Moderate	33 (27.0%)	6 (26.1%)	22 (66.7%)	6 (100%)	0 (0%)	0 (0%)
Severe	35 (28.7%)	1 (4.3%)	27 (77.1%)	1 (100%)	0 (0%)	0 (0%)

^1^Five individuals declined to complete the symptom survey.

**Table 3:** COVID-19 infection duration and concurrent respiratory infection.

					BioFire® testing			
Infection length	# individuals		# NP samples		SARS-CoV-2 (+)		Other respiratory pathogens (+)	
	Cohort 1 (*n* = 18)	Cohort 2 (*n* = 28)	Cohort 1 (*n* = 18)	Cohort 2 (*n* = 71)	Cohort 1	Cohort 2	Cohort 1	Cohort 2
Short	16	11	16	16	*N/A * ^*2*^	14 (87.5%)	0 (0%)	0 (0%)
Long	2	5	2	17	*N/A * ^*2*^	12 (70.6%)	0 (0%)	1 (5.9%)
Recrudescent	*N/A * ^*1*^	12	—	38	—	26 (68.4%)	—	0 (0%)

^1^Unable to determine potential recrudescent infections for cohort 1 due to limited sampling schedule. ^2^Selection criteria for what was considered a short or long infection were dependent on PCR ± status at the first visit.

## Data Availability

The data used to support this study are cited within the article as references 13 and 14 Data are also available upon request from authors Elizabeth P. Ryan (cohort 1) and Gregory D. Ebel (cohort 2).

## Supplementary Materials

Supplementary Materials Supplementary Figure 1: BioFire® Respiratory (RP2.1) panel report from the one NP sample in which concurrent infection was detected.
