# Supplementary material for: The BioFire® RP2.1 Panel Did Not Identify Concurrent Respiratory Virus Infection in Adults with Variable SARS-CoV-2 Disease Severity and Infection Duration
Source: Adv Virol. 2022 Aug 8;2022:1378482. doi: 10.1155/2022/1378482 (PMC9377931; doi:10.1155/2022/1378482)
Supplement: Supplementary Materials — Supplementary Figure 1: BioFire® Respiratory (RP2.1) panel report from the one NP sample in which concurrent infection was detected. [file 1378482.f1.docx]

**Supplementary Figure 1**

**
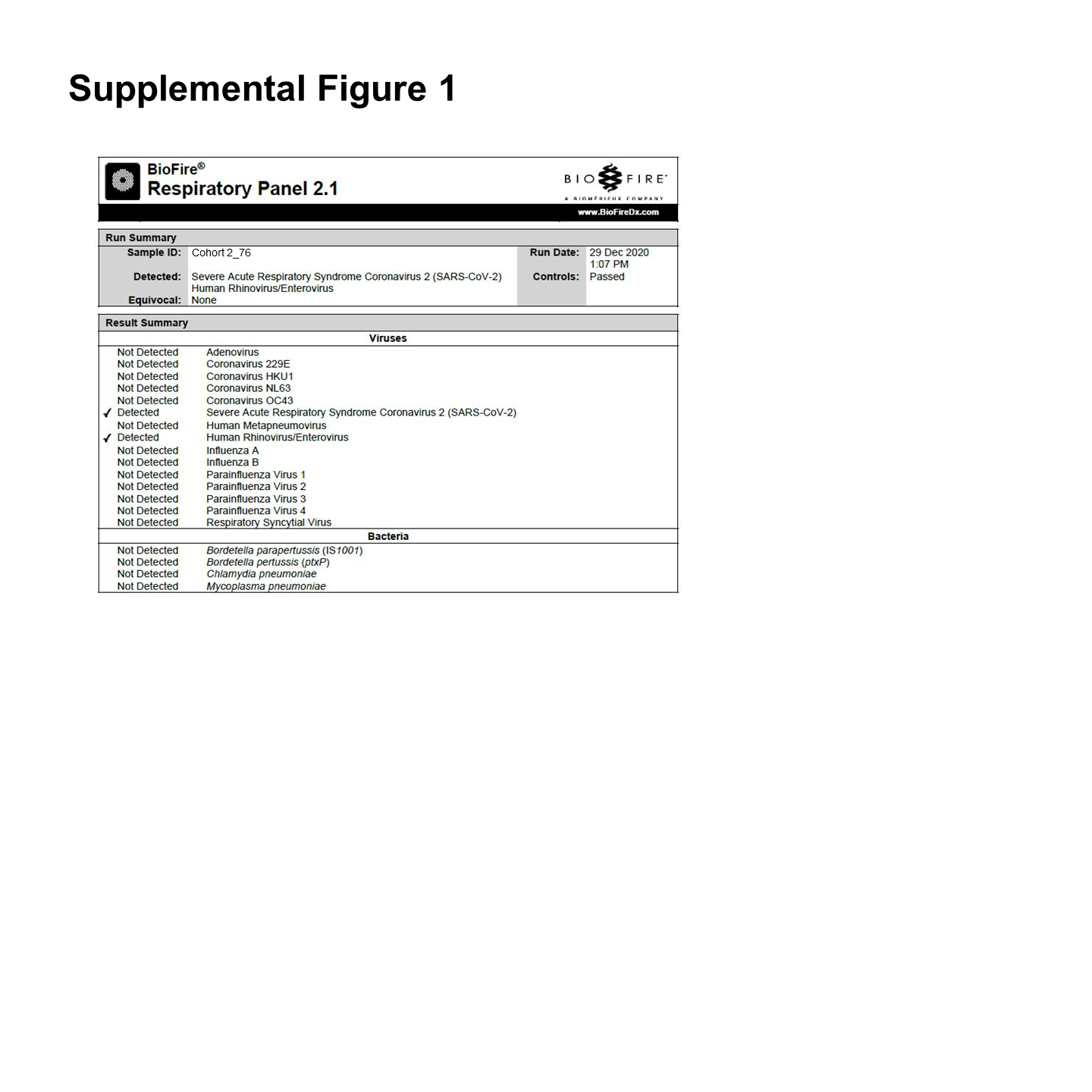
**

**Supplementary Figure Legends**

**Supplementary Figure 1.** ***BioFire® Respiratory (RP2.1) panel report.*** Report generated by the BioFire® RP2.1 panel test from the one NP sample positive for an additional respiratory pathogen. Sample was positive for both SARS-CoV-2 and human rhinovirus/enterovirus.
